# Editorial: The role of sleep in learning and memory

**DOI:** 10.3389/frsle.2025.1652425

**Published:** 2025-08-18

**Authors:** Tony J. Cunningham, Ryan Bottary, Sara E. Alger, Erin J. Wamsley, Dan Denis

**Affiliations:** ^1^Center for Sleep and Cognition, Department of Psychiatry, Beth Israel Deaconess Medical Center, Boston, MA, United States; ^2^Division of Sleep Medicine, Department of Psychiatry, Harvard Medical School, Boston, MA, United States; ^3^Institute for Graduate Clinical Psychology, Widener University, Chester, PA, United States; ^4^Center for Military Psychiatry and Neuroscience, Walter Reed Army Institute of Research, Silver Spring, MD, United States; ^5^Department of Psychology and Program in Neuroscience, Furman University, Greenville, SC, United States; ^6^Department of Psychology, University of York, York, United Kingdom

**Keywords:** memory, learning, interdisciplinary research, dreaming, memory consolidation

Sleep is a universal and essential biological process. Although it is generally understood to play an important role in learning and memory, its exact functions remain a source of ongoing scientific inquiry (Cunningham et al., 2022; Németh et al., 2024). While advances in cognitive and affective neurosciences have identified potential sleep-related mechanisms supporting learning and memory, many questions remain. Does sleep enhance our ability to retain and integrate new information, or can memory consolidation occur independently of sleep? What neurobiological mechanisms allow the sleeping brain to, on one hand, free-up synaptic space for new learning, and on the other hand, strengthen and transform pre-existing memories? What role do dreams play in these processes? These questions are at the heart of this Research Topic, which brings together diverse perspectives to examine behavioral and neurobiological evidence on the importance of sleep for learning, memory, and associated cognitive processes. Research Topics include identifying the mechanisms by which sleep supports memory consolidation, exploring the role of dreaming in cognitive and affective processes, and understanding the consequences of poor sleep for executive functions and emotional regulation. Together, these contributions highlight both the complexity of sleep-related processes and their critical importance for cognition and wellbeing.

Diving into the first theme of this Research Topic, several studies make notable advancements in our understanding of the mechanisms by which sleep supports memory. Using a classic sleep vs. wake design, Reis et al. examined performance on a perceptual learning task after either an afternoon nap or an equivalent period of wakefulness. Participants who napped showed improved memory compared to those who remained awake, demonstrating that sleep supports generalized perceptual learning. Daytime naps have shown to provide similar benefits to memory and cognitive performance as a full night of sleep. To capitalize on this, Fujino et al. tested the effectiveness of napping in a specially designed chair compared to a regular office chair on measures of alertness and cognitive performance. Although post-nap cognitive performance did not differ between the two groups, those who slept in the specialized napping chair reported feeling less sleepy, suggesting a benefit to napping on subjective alertness and vigilance. The temporal coupling of sleep spindles to slow oscillations (SOs) is believed to be a key mechanistic driver of this sleep-associated consolidation (Denis and Cairney, 2024). Supporting these observations, non-invasive closed-loop auditory stimulation of SOs has emerged as an exciting tool to causally manipulate sleep oscillations, and thus potentially affect memory consolidation. Here, Harlow et al. provide a critical meta-analysis of the field, highlighting inconsistencies and methodological issues in the existing literature that will need to be addressed in future work. Further, SO-spindle events are believed to clock hippocampal-reactivation of memory content. Targeted memory reactivation (TMR) paradigms are a non-invasive technique to manipulate these reactivation processes by replaying sounds or odors associated with prior learning. Barner et al. used TMR to reactivate memories of either high or low future relevance. Although highly relevant memories were better retained, TMR had no additional effect, indicating further research may be required to understand the benefits and boundaries of this technique.

Understanding the phenomenology of sleep experience is an important complement to studying its neurophysiology. Dreaming has long been a relatively neglected area, in comparison to the accelerating pace of research progress in other areas of sleep. But recently, the empirical study of dreaming has been reinvigorated by approaching dreams as a downstream result of the reactivation and consolidation of memory in the sleeping brain. Two articles in this Research Topic are emblematic of this fruitful new approach, grounded in the cognitive neuroscience of memory. First, du Plessis and Lipinska report that dream affect predicts memory for emotional information encoded just prior to sleep. Second, Bellaiche et al. use a cognitive neuroscience approach to experimentally manipulate the content of hypnagogic dreaming using directed prompts delivered during pre-sleep wake. Both papers leverage a contemporary understanding of dreaming as a natural extension of waking cognition, molded by experience, memory, emotion and thought in ways similar to waking mind wandering.

The final theme of this Research Topic centers on the premise that we can frequently learn about the function of sleep from its absence. As such, several articles explore the impact of sleep disruption on learning and memory, and on the mechanisms that support these processes. An animal study from Rexrode et al. investigated the impact of acute sleep deprivation on dendritic spines in the mouse amygdala. Given the potential importance of the amygdala in processing emotional and fear memories during sleep, morphological changes would suggest a potential mechanism underlying memory impairments following sleep loss. Acute sleep deprivation led to unique changes in dendritic spines depending on both the region of the amygdala and the morphological subtype of the dendritic spine. The authors conclude that the typical upscaling/downscaling of amygdalar spines during sleep may contribute to aversive memory consolidation. Transitioning to humans, Gilmore et al. used an app-based protocol to collect participants' sleep, stress, and executive function abilities across a 21-day period. Not only did individuals with more regular sleep/wake schedules report fewer acute stress events, but evidence suggested that adaptive executive functioning was preserved, as well. Finally, this Research Topic ends with Wilckens et al. leveraging our understanding of sleep's role in healthy cognitive functioning in the development of a pilot intervention. The authors employed a time-in-bed restriction intervention in older adults and were not only successful at improving sleep quality and efficiency, but also generated a marked increase in slow wave activity—a metric known to be important for sleep-associated consolidation. This study exemplifies how understanding which features of sleep help to optimize our learning and overall functioning can lead to improved targets for clinical interventions.

In sum, spanning animal models to intervention trials, this Research Topic represents a cross-cutting, interdisciplinary approach to extending our knowledge of how sleep affects learning and memory systems. Moreover, this Research Topic represents a microcosm of the work to come in this field. Leveraging sleep's role in learning and memory to optimize human performance and clinical care will require integrating new information collected across the spectrum of human and animal research.
